# Mortality from cardiovascular diseases in sub-Saharan Africa, 1990–2013: a systematic analysis of data from the Global Burden of Disease Study 2013

**DOI:** 10.5830/CVJA-2015-036

**Published:** 2015

**Authors:** George A Mensah, Uchechukwu KA Sampson, Gregory A Roth, Mohammed H Forouzanfar, Mohsen Naghavi, Christopher JL Murray, Andrew E Moran, Valery L Feigin

**Affiliations:** Center for Translation Research and Implementation Science (CTRIS), National Heart, Lung, and Blood Institute, National Institutes of Health, Bethesda, MD, USA; Center for Translation Research and Implementation Science (CTRIS), National Heart, Lung, and Blood Institute, National Institutes of Health, Bethesda, MD, USA; Institute for Health Metrics and Evaluation, University of Washington, Seattle, WA , USA; Institute for Health Metrics and Evaluation, University of Washington, Seattle, WA , USA; Institute for Health Metrics and Evaluation, University of Washington, Seattle, WA , USA; Institute for Health Metrics and Evaluation, University of Washington, Seattle, WA , USA; Division of General Medicine, Columbia University Medical Center, New York, NY, USA; Faculty of Health and Environmental Sciences, National Institute for Stroke and Applied Neurosciences, Auckland University of Technology, Auckland, New Zealand

**Keywords:** cardiovascular diseases, sub-Saharan Africa, epidemiology, mortality rate, global burden of disease, developing countries

## Abstract

**Background:**

Cardiovascular disease (CVD) has been the leading cause of death in developed countries for most of the last century. Most CVD deaths, however, occur in low- and middle-income, developing countries (LMICs) and there is great concern that CVD mortality and burden are rapidly increasing in LMICs as a result of population growth, ageing and health transitions. In sub-Saharan Africa (SSA), where all countries are part of the LMICs, the pattern, magnitude and trends in CVD deaths remain incompletely understood, which limits formulation of data-driven regional and national health policies.

**Objective:**

The aim was to estimate the number of deaths, death rates, and their trends for CVD causes of death in SSA, by age and gender for 1990 and 2013.

**Methods:**

Age- and gender-specific mortality rates for CVD were estimated using the Global Burden of Disease (GBD) 2010 methods with some refinements made by the GBD 2013 study to improve accuracy. Cause of death was estimated as in the GBD 2010 study and updated with a verbal autopsy literature review and cause of death ensemble modelling (CODEm) estimation for causes with sufficient information. For all quantities reported, 95% uncertainty intervals (UIs) were also computed.

**Results:**

In 2013, CVD caused nearly one million deaths in SSA, constituting 38.3% of non-communicable disease deaths and 11.3% of deaths from all causes in that region. SSA contributed 5.5% of global CVD deaths. There were more deaths in women (512 269) than in men (445 445) and more deaths from stroke (409 840) than ischaemic heart disease (258 939). Compared to 1990, the number of CVD deaths in SSA increased 81% in 2013. Deaths for all component CVDs also increased, ranging from a 7% increase in incidence of rheumatic heart disease to a 196% increase in atrial fibrillation. The age-standardised mortality rate (per 100 000) in 1990 was 327.6 (CI: 306.2–351.7) and 330.2 (CI: 312.9–360.0) in 2013, representing only a 1% increase in more than two decades.

**Conclusions:**

In SSA, CVDs are neither epidemic nor among the leading causes of death. However, a significant increase in the number of deaths from CVDs has occurred since 1990, largely as a result of population growth, ageing and epidemiological transition. Contrary to what has been observed in other world regions, the age-adjusted mortality rate for CVD has not declined. Another important difference in CVD deaths in SSA is the predominance of stroke as the leading cause of death. Attention to aggressive efforts in cardiovascular health promotion and CVD prevention, treatment and control in both men and women are warranted. Additionally, investments to improve directly enumerated epidemiological data for refining the quantitation of risk exposures, death certification and burden of disease assessment will be crucial.

## Abstract

Cardiovascular disease (CVD), principally ischaemic heart disease and stroke, constitute the leading cause of global mortality, and accounted for 17.3 million deaths worldwide in 2013.^1^ In high-income, developed countries, CVDs have been the leading cause of death throughout most of the last century. In the United States, for example, heart disease has been the leading cause of death every year since 1918.^2^ Currently, however, most CVD deaths occur in low- and middle-income countries (LMIC) and there is growing concern that an epidemic of CVDs is emerging in these countries that must be prevented.^3^,^4^ The World Health Organisation (WHO) and the United Nations have also called attention to a rising burden of CVD and other non-communicable diseases and the need for aggressive measures to forestall this epidemic in these countries^5^,^6^

In sub-Saharan Africa (SSA), where all countries are part of the developing world, the magnitude of and trends in CVD deaths remain incompletely understood. The African regional office of the WHO has stated that CVDs are ‘increasing rapidly in Africa, and it is now a public health problem throughout the African region’.^7^ However, a systematic analysis of estimates for CVD mortality in the Global Burden of Disease (GBD) 2010 study showed no significant rise in age-standardised mortality rates for CVD in SSA for the period of 1990–2010.^8^,^9^ Similarly, recent data from the INDEPTH Health and Demographic Surveillance System show little evidence of NCD mortality rates increasing over time.^10^,^11^ However, data on CVDs are limited in the region,^12^-^14^ and novel methods are required in order to make meaningful estimates.

Clarifying the epidemiology of CVD in SSA is essential for the formulation of regional and national health policies. Accordingly, we explored as a primary objective, estimates of the number of deaths, age-standardised and age- and gender-specific mortality rates, and their trends in SSA, by age and gender, for the period 1990–2013 for total CVD, rheumatic heart disease (RHD), ischaemic heart disease (IHD), cerebrovascular disease (including ischaemic and haemorrhagic stroke), hypertensive heart disease, cardiomyopathy and myocarditis, atrial fibrillation and flutter, aortic aneurysm, peripheral arterial disease (PAD) and endocarditis. These data for SSA were also compared to data for developing and developed countries.

## Methods

Age and gender year-specific mortality rates for CVD were estimated using the methods as published in the GBD 2013 study.^1^ In brief, the GBD 2013 study collected all available data on mortality, including vital registration and verbal autopsy. Raw data were corrected to account for outliers and non-specific causes of death (i.e. ‘garbage’ codes).

Modelling was performed using a custom ensemble-model approach (CODem) to estimate deaths for each country, including countries without data.^15^ CODem employs Gaussian process regression and spatio-temporal modelling, as well as cardiovascular-specific covariates, such as systolic blood pressure, to produce consistent estimates.1 Estimates were adjusted to fit an envelope of all-cause mortality and all-cardiovascular mortality to ensure that no strata contained more deaths that occurred for any of its parent categories.

For SSA, mortality data for the years 1980–2011 were used from Madagascar, Ethiopia, Mauritius, Seychelles, South Africa, Zambia, Mozambique, Kenya, Tanzania, Burkino Faso, Zimbabwe, Mali and Ghana. For all quantities reported, 95% uncertainty intervals (UIs) were also computed using 1 000 draws from the posterior distribution of each age–gender–country–year-specific set of estimates. Death numbers from each country and each cause were summed to produce estimates for the entire region of SSA.

## Results

As shown in Table 1, CVDs caused nearly one million deaths in SSA in 2013. The number of deaths in women (512 269) exceeded those in men (445 445) for total CVDs and also for all cardiovascular causes of death except ischaemic heart disease, aortic aneurysms and peripheral vascular disease. There were more deaths from stroke (409 840) than ischaemic heart disease (258 939). Compared to 1990, CVD deaths increased 81% in 2013. Similarly, deaths for all component CVDs also increased, ranging from a 7% increase in rheumatic heart disease to a 196% increase in atrial fibrillation. The age-standardised mortality rate (per 100 000) for total CVD in 1990 was 327.6 (CI: 306.2–351.7) and 330.2 (CI: 312.9–360.0) in 2013, representing a 1% increase.

**Table 1:** Total number of deaths and age-standardised mortality rates for component cardiovascular causes of death in 1990 and 2013 and the respective percentage changes

*Cause*	*Number of deaths, 1990*	*95% UI*	*Number of deaths, 2013*	*95% Ul*	*% Change*	*Age-standardized death rate (per 100 000), 1990*	*95% UI*	*Age-standardized death rate (per 100 000), 1990*	*95%UI*	*% Change*
Ischaemic heart disease	138 308	(116 618 – 153 645)	258 939	(232 158 – 305 680)	87	91.4	(76.9 – 101.7)	92.9	(82.8 – 110.2)	2
Ischaemic stroke	101 040	(77 903 – 117 660)	206 439	(139 860 – 242 225)	104	75.0	(57.2 – 87.5)	81.5	(55.0 – 95.7)	9
Hemorrhagic stroke	125 603	(103 055 – 147 517)	203 401	(173 620 – 262 418)	62	72.2	(57.1 – 87.6)	64.7	(54.0 – 87.5)	–10
Hypertensive heart disease	37 525	(29 485 – 49 443)	86 035	(62 970 – 111 978)	129	26.8	(21.0 – 36.5)	32.8	(24.2 – 44.0)	22
Cardiomyopathy	28 917	(23 557 – 36 082)	53 742	(44 926 – 65 634)	86	12.7	(10.6 – 17.0)	14.5	(11.9 – 18.2)	14
Rheumatic heart disease	23 625	(17 644 – 31 608)	25 239	(20 478 – 40 444)	7	10.3	(7.5 – 13.7)	6.5	(5.3 – 10.1)	–37
Atrial fibrillation	414	(331 – 509)	1 227	(959 – 1 558)	196	0.4	(0.3 – 0.5)	0.6	(0.5 – 0.8)	50
Aortic aneurysm	5 150	(3 370 – 6 714)	9 854	(7 809 – 12 840)	91	3.3	(2.2 – 4.3)	3.4	(2.7 – 4.5)	3
Peripheral vascular disease	469	(371 – 580)	1 338	(1 122 – 1 618)	185	0.4	(0.3 – 0.5)	0.6	(0.5 – 0.7)	50
Endocarditis	9 622	(6 339 – 15 825)	13 868	(10 967 – 18 524)	44	4.7	(3.0 – 8.6)	3.7	(2.9 – 5.3)	–21
Other cardiovascular diseases	59 206	(48 291 – 74 859)	98 632	(77 904 – 138 971)	67	30.3	(24.8 – 41.1)	29.1	(22.7 – 42.8)	–4
Total cardiovascular diseases	529 880	(492 351 – 568 410)	958 713	(909 427 – 1 049 606)	81	327.6	(306.2 – 351.7)	330.2	(312.9 – 360.0)	1

As previously demonstrated, SSA experiences the world’s lowest IHD death rates, and IHD ranks below stroke as a leading cause of CVD death in the region.12 On average, SSA experienced no significant change in age-standardised IHD mortality rate between 1990 and 2013, however, likely due to aging and growth of the SSA population, the number of IHD deaths increased by 87% over the same interval (Table 1).

**Fig. 1.**
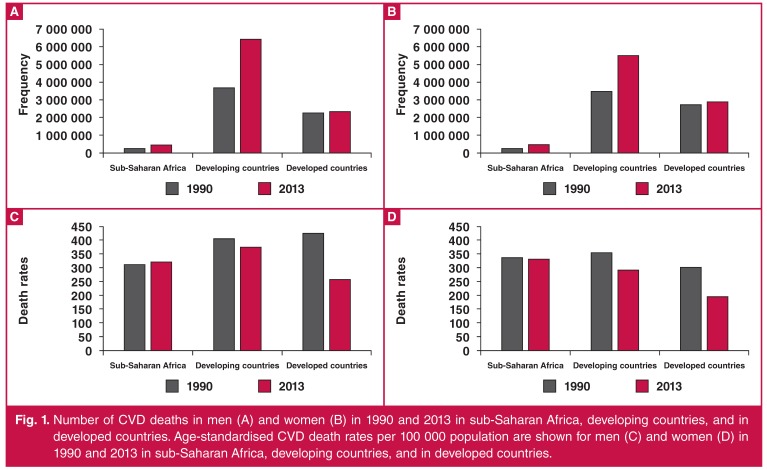
Number of CVD deaths in men (A) and women (B) in 1990 and 2013 in sub-Saharan Africa, developing countries, and in developed countries. Age-standardised CVD death rates per 100 000 population are shown for men (C) and women (D) in 1990 and 2013 in sub-Saharan Africa, developing countries, and in developed countries.

As shown in Fig. 1 for both men and women, the number of CVD deaths in SSA was substantially lower than that seen for either the developed or developing countries. Contrary to the pattern seen for developed and developing countries (as a whole), the age-standardised mortality rate for CVD in both men and women in SSA did not decline during the period from 1990 to 2013 (Fig. 1). In fact, the age-standardised mortality rate for women in SSA, which was lower than the corresponding rate in women in developing countries in 1990, is now higher than the rate seen for women in developing countries, and substantially higher than the corresponding rates for both men and women in the developed world (Fig. 1).

The number of deaths from stroke in SSA nearly doubled from 1990 to 2013, but overall age-adjusted stroke mortality rates decreased by 1% (increased by 9% for ischaemic stroke and decreased by 10% for haemorrhagic stroke). The increase in number of stroke deaths in SSA was particularly noticeable for ischaemic stroke (104% increases in 2013 compared to 1990), although age-adjusted stroke mortality rates in SSA regions were one of the lowest in LMIC and only slightly higher than that in developed countries, compared with GBD 2010 stroke mortality estimates. Although the majority of deaths from stroke in SSA in 1990 were due to haemorrhagic strokes (55%), in 2013 the proportional frequency of deaths from haemorrhagic stroke was slightly lower (49.6%) than that from ischaemic stroke. Compared to GBD 2010 stroke mortality estimates, the mean age at death from stroke in SSA was the lowest among all LMIC.

In 1990 the total number of deaths from PAD was 469 (CI: 371–580) compared with 1 338 (CI: 1 122–1 618) in 2013, representing a 185% increase. There were 277 (CI: 204–366) deaths due to PAD among men, which was higher than the 192 (CI: 145–271) deaths observed among women. Similarly, in 2013, the number of PAD deaths among men was 728 (CI: 578–902), which was also higher than the 610 (CI: 480–817) deaths observed among women. The combined age-standardised death rates per 100 000 were 0.4 (CI: 0.3–0.5) and 0.6 (CI: 0.5–0.7) in 1990 and 2013, respectively, representing a 50% increase during the 23-year period. The age-standardised death rates for men were 0.5 (CI: 0.3–0.6) in 1990 and 0.6 (CI: 0.5–0.8) in 2013, which represents a 20% increase. However, women had a 66% increase in age-standardised death rates, as evidenced by the change from 0.3 (CI: 0.2–0.5) in 1990 to 0.5 (CI: 0.4–0.7) in 2013.

The total number of deaths from atrial fibrillation was 414 (331–509) in 1990, compared with 1 227 (CI: 959–1 558) in 2013, representing an increase of 196%. However, the age-standardised death rates (per 100 000) increased by 50% during the study period from 0.4 (CI: 0.3–0.5) in 1990 to 0.6 (CI: 0.5–0.8) in 2013. In 1990 there were 148 (CI: 106–193) deaths due to atrial fibrillation among men, which was less than the 266 (CI: 201–347) deaths observed among women. In 2013 there was a similar pattern of fewer AFIB deaths in men compared with women: 378 (CI: 295–490) vs 848 (CI: 605–1 170). The age-standardised death rates for men were 0.3 (CI: 0.2–0.4) in 1990 and 0.4 (CI: 03–0.5) in 2013. The corresponding rates for women were 0.5 (CI: 0.3–0.7) and 0.7 (CI: 0.5–1.0), respectively.

The mortality rate from PAD in SSA has increased over the last 23 years. Furthermore, the relative increase in PAD mortality rate among women has been more dramatic than among men. Similar findings are noted for AFIB, wherein we actually observed a higher number of deaths and age-standardised death rates among women compared with men.

## Discussion

The most prominent finding in this study was that the age-standardised mortality rate for CVD has not declined in SSA, in sharp contrast to the dramatic declines that have been documented in other world regions, especially in the high-income, developed world. It is of concern that the age-standardised mortality rate for CVD in SSA women, which was lower than the corresponding rate in women in developing countries in 1990, is now higher than in developing countries, and substantially higher than corresponding rates in the developed world. In fact, a previous analysis of the GBD 2013 data on demographic and epidemiological drivers of global CVD mortality suggested that age-specific death rates for western sub-Saharan Africa may have increased.^16^ These findings have significant implications for effective prevention and treatment of CVD in SSA.

A second important observation from this study was that although most CVD deaths occur in developing countries, the overall number of CVD deaths in SSA is substantially lower than seen in the rest of the developed and developing world, and amounts to 5.5% of global CVD deaths. In addition, these CVD deaths in SSA constitute 38.3% of non-communicable disease deaths and 11.3% of deaths from all causes in that region.^1^ Therefore the assertion that CVD deaths represent an emerging epidemic may be unwarranted. Importantly, however, the approximately one million deaths in 2013 in SSA represent a near doubling of the deaths a decade earlier. Roth *et al.*^16^ have showed that population growth and ageing accounted for the increase in the number of global deaths due to CVD between 1990 and 2013, despite an overall decrease in age-specific death rates for most regions.^16^

A careful analysis of the data from SSA to determine the role that population growth, ageing and health transitions play in these deaths is needed. Uniformly however, component CVD deaths increased by a range from 7% for RHD to nearly a three-fold increase in atrial fibrillation and peripheral arterial disease. The good news from the data is the relative decline in the age-standardised mortality rates for RHD, haemorrhagic stroke and endocarditis.

This study has several limitations. Some cardiovascular causes of death are less commonly reported as an underlying cause of death. These diseases include rheumatic heart disease, atrial fibrillation, peripheral vascular disease, endocarditis and aortic aneurysms. Changes over time in the detection and reporting of these conditions most likely reflect not just epidemiological changes but changes in diagnostics and the ways that these diagnoses are attributed to deaths by physicians. Rheumatic heart disease-related mortality may be particularly difficult to attribute as a cause of death, which increases uncertainty for estimates of this condition.

Importantly, data sources on disease burden in SSA are among the most limited in the world, despite several comprehensive networks for data collection related to maternal and child mortality. Unfortunately a functioning vital records system can only be built in a society with a functioning primary health system that provides broad access – a concept that remains a serious challenge for most of SSA within the foreseeable future. Verbal autopsy and sample vital registration will likely expand as countries expand their investment in health system infrastructure.

Efforts should focus on improving comparability across countries and regions within SSA. Increased attention to non-communicable diseases will help highlight the important role of routine surveillance, rather than one-off research studies, as an important source of descriptive statistics related to CVD in SSA.

## Conclusions

SSA has seen no significant decline in age-standardised CVD mortality rates, whereas these rates continue to fall dramatically in most of the high- and middle-income world. Without further investment in the prevention and treatment of CVD and other NCDs and their risk factors in SSA, the continent risks being left behind at a time when improved detection, prevention, treatment and control of these diseases and risk factors are leading to longevity and improved quality of life in other world regions. These decisions should be informed ideally by reliably accurate, directly enumerated data, rather than estimates such as those presented in this study.

The relative lack of directly enumerated epidemiological data, coupled with the absence of vital registration systems in 42 of the 46 SSA countries, presents major challenges in mortality and burden of disease data for this region. The estimates of mortality presented here must therefore be interpreted with caution. Nevertheless, we found no evidence to support a rapidly rising epidemic or an impending pandemic of CVD in SSA. However, the consistency of the directional changes in CVD deaths, disability and risk-factor trends observed in GBD 2010, and the mortality trends seen in GBD 2013, together with the young average age at time of death from CVDs in SSA, compel attention to aggressive efforts at CVD risk-factor prevention, treatment and control in both women and men. Coordinated partnerships between ministries of health, other government agencies, non-governmental organisations, and the private sector will be essential in order to mount a targeted response to the observed challenges.

Interventions at the individual and population levels as well as improved systems of healthcare will be required. In addition, investments to improve local-level directly enumerated epidemiological data and refinement of the quantitation of risk exposure, death certification and burden of disease assessment will be crucial. Further research is needed to identify ideal dissemination and implementation strategies for CVD risk reduction in the SSA setting.

Until then, finding ways to implement interventions that are feasible, affordable and acceptable to the local population and are appropriate to implement in low-resource settings for the prevention and control of CVD in Africa should be a priority.^17^ This strategy is particularly relevant for the clinical and public health approaches for addressing hypertension, diabetes, unhealthy diet, physical inactivity and tobacco use. Improving access to directly enumerated epidemiological data for the region would also go a long way towards appropriately informed healthcare policy and practice.
